# microRNA-122 Abundance in Hepatocellular Carcinoma and Non-Tumor Liver Tissue from Japanese Patients with Persistent HCV versus HBV Infection

**DOI:** 10.1371/journal.pone.0076867

**Published:** 2013-10-09

**Authors:** Carolyn Spaniel, Masao Honda, Sara R. Selitsky, Daisuke Yamane, Tetsuro Shimakami, Shuichi Kaneko, Robert E. Lanford, Stanley M. Lemon

**Affiliations:** 1 Departments of Medicine and Microbiology & Immunology and the Lineberger Comprehensive Cancer Center, the University of North Carolina at Chapel Hill, Chapel Hill, North Carolina, United States of America; 2 Department of Microbiology and Immunology, University of Texas Medical Branch, Galveston, Texas, United States of America; 3 Department of Gastroenterology, Kanazawa University Graduate School of Medicine, Takara-Machi, Kanazawa, Japan; 4 Department of Genetics, the University of North Carolina at Chapel Hill, Chapel Hill, North Carolina, United States of America; 5 Department of Virology and Immunology, Texas Biomedical Research Institute, San Antonio, Texas, United States of America; Inserm, U1052, UMR 5286, France

## Abstract

Mechanisms of hepatic carcinogenesis in chronic hepatitis B and hepatitis C are incompletely defined but often assumed to be similar and related to immune-mediated inflammation. Despite this, several studies hint at differences in expression of miR-122, a liver-specific microRNA with tumor suppressor properties, in hepatocellular carcinoma (HCC) associated with hepatitis B virus (HBV) versus hepatitis C virus (HCV) infection. Differences in the expression of miR-122 in these cancers would be of interest, as miR-122 is an essential host factor for HCV but not HBV replication. To determine whether the abundance of miR-122 in cancer tissue is influenced by the nature of the underlying virus infection, we measured miR-122 by qRT-PCR in paired tumor and non-tumor tissues from cohorts of HBV- and HCV-infected Japanese patients. miR-122 abundance was significantly reduced from normal in HBV-associated HCC, but not in liver cancer associated with HCV infection. This difference was independent of the degree of differentiation of the liver cancer. Surprisingly, we also found significant differences in miR-122 expression in non-tumor tissue, with miR-122 abundance reduced from normal in HCV- but not HBV-infected liver. Similar differences were observed in HCV- vs. HBV-infected chimpanzees. Among HCV-infected Japanese subjects, reductions in miR-122 abundance in non-tumor tissue were associated with a single nucleotide polymorphism near the IL28B gene that predicts poor response to interferon-based therapy (TG vs. TT genotype at rs8099917), and correlated negatively with the abundance of multiple interferon-stimulated gene transcripts. Reduced levels of miR-122 in chronic hepatitis C thus appear to be associated with endogenous interferon responses to the virus, while differences in miR-122 expression in HCV- versus HBV-associated HCC likely reflect virus-specific mechanisms contributing to carcinogenesis. The continued expression of miR-122 in HCV-associated HCC may signify an important role for HCV replication late in the progression to malignancy.

## Introduction

Globally, liver cancer is the fifth and seventh most common malignancy in men and women, respectively, and the third most deadly [1]. Most (85-95%) of these cancers are hepatocellular carcinoma (HCC) [2], and many are associated with persistent intrahepatic infections with hepatitis C virus (HCV) or hepatitis B virus (HBV) [2,3]. Although the total cancer death rate decreased within the United States by over 1.5% between 2001-2007, deaths due to liver cancer increased by 50% among males and by 29% in females [4]. These changes in the incidence of HCC are largely due to increases in HCV-associated malignancy. Similarly, while HBV infection historically has been the major risk factor underlying development of HCC in Asia, in Japan it has been supplanted in recent decades by HCV infection [5].

The exact mechanisms underlying HCV- and HBV-associated malignancy are unknown [6,7]. Chronic infections with either virus may result in cirrhosis, which alone is a major risk factor for liver cancer [2]. However, there may also be virus-specific mechanisms at work. While immune-mediated mechanisms are both necessary and sufficient for the development of HBV-related cancer in murine models, liver cancer arises in the absence of inflammation in HCV- transgenic mice [8,9]. Moreover, some HCV proteins may interact with host tumor suppressors and possibly impair cellular responses to DNA damage [10]. If virus-specific mechanisms of oncogenesis are important in the development of HCC, it is reasonable to anticipate that the pathways leading to HCV- and HBV-associated cancer might differ, possibly leaving distinguishing genetic or epigenetic marks in the tumors that arise. If so, understanding these differences would be important for biomarker discovery, and potentially design of preventative and therapeutic interventions.

Here, we describe a study that was aimed at determining whether the abundance of microRNA-122 (miR-122) is different in liver cancer arising in patients with chronic HCV infection compared to cancers arising in the context of chronic HBV infection. Mature microRNAs (miRNAs) are 20-23 nucleotides in length and encoded either by microRNA genes or from within conventional protein-coding genes. They act generally by binding to specific sites within the 3’ untranslated region (3’ UTR) of cellular mRNAs, to which they recruit RNA-induced silencing complexes (RISC) that repress translation and destabilize the mRNA [11–13]. miR-122 is a liver-specific miRNA that accounts for the majority of miRNAs in hepatocytes [14]. It regulates a large number of genes within the liver [15], and has several tumor suppressor-like properties [16,17]. Importantly, miR-122 is a crucial host factor for HCV replication, binding to the 5’ untranslated RNA segment of the viral genome, physically stabilizing it, and promoting viral protein expression [18–20].

Because of its liver-specific nature and tumor suppressor-like qualities [16,17], it is of interest to know whether miR-122 expression is altered in liver cancers. Prior studies investigating miR-122 expression in liver cancers have produced conflicting results, particularly as related to the underlying viral causes of cancer. Two early studies suggest that miR-122 abundance is generally reduced in HCC [21,22]. However, Hou et al. [23] reported that miR-122 expression was maintained in both HBV- and HCV-associated cancer, while Varnholt et al. [24] reported that miR-122 levels were increased significantly in HCV-associated cancers when compared to non-cancerous tissue. Coulouarn et al. [25] reported higher miR-122 expression levels in HCV- versus HBV-associated cancers. To some extent, these conflicting results may reflect different patient populations, or possibly methodologic differences, not only in the measurement of miR-122 abundance but also in how miR-122 abundance was compared across tissue samples.

In an effort to resolve this controversy, we conducted a comprehensive analysis of miR-122 expression in liver cancers arising in a genetically homogenous group of Japanese patients. Using a highly accurate, miR-122-specific quantitative reverse-transcription, polymerase chain reaction (qRT-PCR) assay, and paying particular attention to how miR-122 measurements are compared between tissue samples, we show that miR-122 expression is significantly reduced in HBV-associated HCC but not in most HCV-associated cancers. We also demonstrate that miR-122 abundance is reduced in non-tumor HCV-infected liver in association with increased expression of interferon (IFN)-stimulated genes (ISGs).

## Materials and Methods

### Ethics statement

Liver tissue was obtained from Japanese patients undergoing surgical resection of liver cancer (primary or metastatic) at the Liver Center of Kanazawa University Hospital (Kanazawa, Japan). All subjects provided written informed consent for participation in the study, and tissue acquisition procedures were approved by the ethics committee of Kanazawa University under a protocol entitled "Gene expression analysis of peripheral blood cells and liver in patients with liver and gastrointestinal cancers". Archived liver tissue and serum samples were collected prior to December 15, 2011 from chimpanzees housed and cared for at the Southwest National Primate Research Center (SNPRC) of the Texas Biomedical Research Institute in accordance with the Guide for the Care and Use of Laboratory Animals. All protocols were approved by the Institutional Animal Care and Use Committee. SNPRC is accredited by the Association for Assessment and Accreditation of Laboratory Animal Care (AAALAC) International and operates in accordance with the NIH and U.S. Department of Agriculture guidelines and the Animal Welfare Act.

### Human subjects and tissue samples

Paired samples of HCC and non-tumor liver tissue were obtained from Japanese patients undergoing surgical resection of HCC at the Liver Center of Kanazawa University Hospital (Kanazawa, Japan). Non-infected ‘normal’ liver tissue was similarly collected from patients undergoing resection of metastases of non-hepatic primary cancers. Patients were categorized as HCV-infected by the presence of HCV RNA (COBAS Ampli-Prep/COBAS TaqMan System) and absence of hepatitis B surface antigen (HBsAg) in serum or plasma at the time of surgery, while HBV infection was defined by the presence of HBsAg and absence of anti-HCV antibodies. HCC was categorized according to the degree of cellular differentiation, while fibrosis and inflammation in non-tumor tissue from HBV- and HCV-infected patients were compared after scoring each [26,27]. The IL28B genotype of study subjects with HCV infection was defined at the rs8099917 locus as described previously [28].

### Chimpanzee care and sample collection

We studied archived liver tissue and serum samples collected prior to December 15, 2011 from chimpanzees housed and cared for at the Southwest National Primate Research Center (SNPRC) of the Texas Biomedical Research Institute. At the time samples were obtained, animals considered to be non-infected (‘normal’) were negative for HBV and HCV markers; HBV infection was defined as the presence of serum HBsAg, and HCV infection by the presence of HCV RNA detectable in sera by RT-PCR.

### Small RNA quantitation in human samples

Human tissue samples were stored in liquid nitrogen until processed for RNA extraction. Approximately 1 mg of tissue was ground using a tissue homogenizer and total RNA isolated using the mirVana miRNA isolation kit (Ambion). Liver RNA samples were subsequently stored at -80°C or on dry ice during shipment. The quality of the isolated RNA (RIN score) was assessed using an Agilent 2100 Bioanalyzer (Agilent RNA 6000 Nano Kit, Agilent Technologies) [29]. Quantification of miR-122, miR-191, Let-7a, miR-24, and the small nuclear RNA (snRNA) U6 was carried out by quantitative reverse-transcription, polymerase chain reaction (qRT-PCR) in a two-step process. RNA (12.5 ng) was reversed transcribed in a 10 µl reaction mix using reagents provided with the Universal cDNA Synthesis kit (Exiqon) and the manufacturer’s recommended procedure. Quantitative PCR was carried out subsequently with the SYBR Green Master Mix Kit (Exiqon), mixed locked-nucleic acid primer sets specific for each miRNA or snRNA (Exiqon), and the CFX96 PCR System (Bio-Rad). Results are presented as relative copy number normalized to total RNA. Alternatively, absolute miR-122 copy numbers were estimated using serial dilutions of single-stranded synthetic miR-122 (Dharmacon) as a standard.

### miR-122 and HCV RNA quantitation in chimpanzee samples

Total RNA was extracted from serum and liver using RNA Bee (Leedo Medical Labs, Houston, TX), chloroform extraction and isopropanol precipitation. Detection of miR-122 was performed using primers and probes for miR-122 included in the ABI TaqMan assay (Cat No. 4373151) and the ABI TaqMan microRNA Reverse Transcription Kit (Cat No. 4366596). The RT reaction was performed with 5 ng of total cell RNA, and the PCR amplification was performed with one-tenth of the resulting cDNA. The RT reaction was performed at 16°C for 30 min, followed by 42°C for 30 min, and 85°C for 5 min. The TaqMan Universal PCR Master Mix with no AmpErase UNG was used for PCR amplification with reaction conditions of 95°C for 10 min followed by 40 cycles at 95°C for 15 sec and 60°C for 1 min. A standard curve was generated using a synthetic RNA equivalent to mature miR-122. HCV viral RNA levels in the serum and liver were determined using a real-time, quantitative RT-PCR (TaqMan) assay detecting sequences in the viral 5’ noncoding RNA using an ABI 7500 sequence detector (PE Biosystems, Foster City, CA) as previously described [30]. Synthetic HCV RNA was used to generate a standard curve for determination of genome equivalents. The forward primer was from nucleotide 149 to 167 (5’- tgcggaaccggtgagtaca-3’), the reverse primer was from nucleotide 210 to 191 (5’-cgggtttatccaagaaagga-3’) and the probe was from nucleotide 189 to 169 (5’-ccggtcgtcctggcaattccg-3’) in the 5’ NCR of HCV.

### Affymetrix array analysis

Human RNA samples were subjected to high-density oligonucleotide microarray analysis as described previously [28]. In brief, cDNA amplified using the WT-Ovation Pico RNA Amplification System (NuGen, San Carlos, CA, USA) was used for fragmentation and biotin labeling with the FL-Ovation cDNA Biotin Module V2 (NuGen). Biotin-labeled cDNA suspended in hybridization cocktail (NuGen) was hybridized to Affymetrix U133 Plus 2.0 GeneChips, followed by labeling with streptavidin-phycoerythrin. Probe hybridization was determined using a GeneChip Scanner 3000 (Affymetrix) and analyzed using GeneChip Operating Software 1.4 (Affymetrix).

### Statistical analysis

Statistical analyses were carried out using Prism V software (Graphpad Software, Inc). The paired t test was used for comparison of results arising from groups of paired tissue specimens (HCC versus non-tumor tissue), while the unpaired t test or Mann-Whitney test was used for comparisons between groups of unrelated tissues (e.g., HBV versus HCV infection). Nonparametric analysis of the correlation between miR-122 and ISG expression levels was done by the Spearman method. Other statistical tests were as described in the text.

## Results

### miR-122 abundance in HCV- versus HBV-associated liver cancer

**Figure 1:**
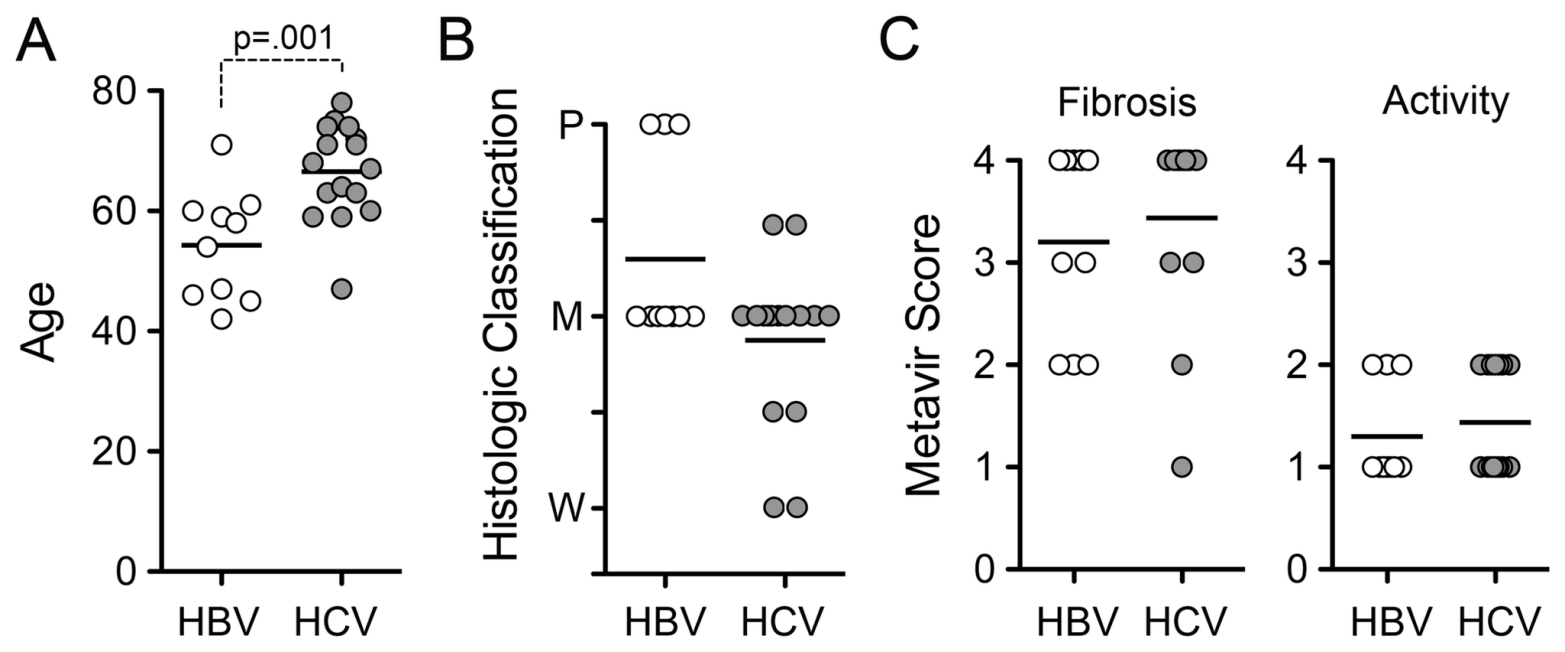
Age, histological classification of tumors, and scoring of non-tumor tissue for inflammation and fibrosis. (**A**) Age of subjects from whom HBV- and HCV-associated HCC and paired non-tumor samples were obtained. (**B**) Histological classification of tumors: W = well differentiated, M = moderately differentiated, P = poorly differentiated. (**C**) Individual scores for fibrosis and inflammatory activity in non-tumor tissue. Bars represent mean values. See also Table 1.

We measured miR-122 abundance in paired tumor and non-tumor tissues collected from 26 patients undergoing surgical resection of HCC: 16 with concomitant chronic HCV infection, and 10 infected with HBV. The age, gender, histological classification of HCC, and fibrosis score of non-tumor tissues are shown in Figure 1 (see also Table 1). Subjects infected with HCV (predominantly genotype 1b) were approximately one decade older than those with HBV infection (66.6 ± 8.0 s.d. versus 54.3 ± 9.1 s.d. years, p=0.001, Figure 1A), consistent with previous studies indicating that HCC is generally diagnosed at an earlier age in HBV-infected Japanese patients [31]. There were no significant differences in the histological classification of HCC or scores for fibrosis or inflammatory activity in non-tumor tissues between the two groups (Figure 1B and C, and Table 1). There were more females among those with HCV infection (10 male and 6 female) than HBV (9 male and 1 female), but this difference did not achieve statistical significance (Chi square test with Yate’s correction).

**Table 1:** Characteristics of Study Subjects.

	HCV (n = 16)	HBV (n=10)	Non-infected (n=9)
Mean Age (years)	66.6 ± 8.0 s.d.	54.3 ± 9.1	60.1 ± 14.3
Gender (M/F)	10M/6F	9M / 1F	5M / 4F
HCV Genotype			
*1a*	0	n/a	n/a
*1b*	14		
*2*	2		
*3*	0		
Fibrosis Stage	n (%)	n (%)	n (%)
0	0 (0)	0 (0)	9 (100)
1	1 (6)	0 (0)	0 (0)
2	1 (6)	3 (30)	0 (0)
3	4 (25)	2 (20)	0 (0)
4	10 (63)	5 (50)	0 (0)
Inflammation	n (%)	n (%)	n (%)
0	0 (0)	0 (0)	9 (100)
1	9 (56)	7 (70)	0 (0)
2	7 (44)	3 (30)	0 (0)
3	0 (0)	0 (0)	0 (0)
4	0 (0)	0 (0)	0 (0)
HCC Histologic Differentiation	n (%)	n (%)	
*Well*	2 (13)	0 (0)	n/a
*Moderate-Well*	2 (13)	0 (0)	
*Moderate*	10 (63)	7 (30)	
*Poor-Moderate*	2 (13)	0	
*Poor*	0 (0)	3 (30)	
IL28B genotype (rs8099917)	n (%)		
*TT*	9 (56)	n.d.	n.d.
*TG*	7 (44)		
*GG*	0 (0)		

n/a = “not applicable”; n.d. = “not done”

**Figure 2:**
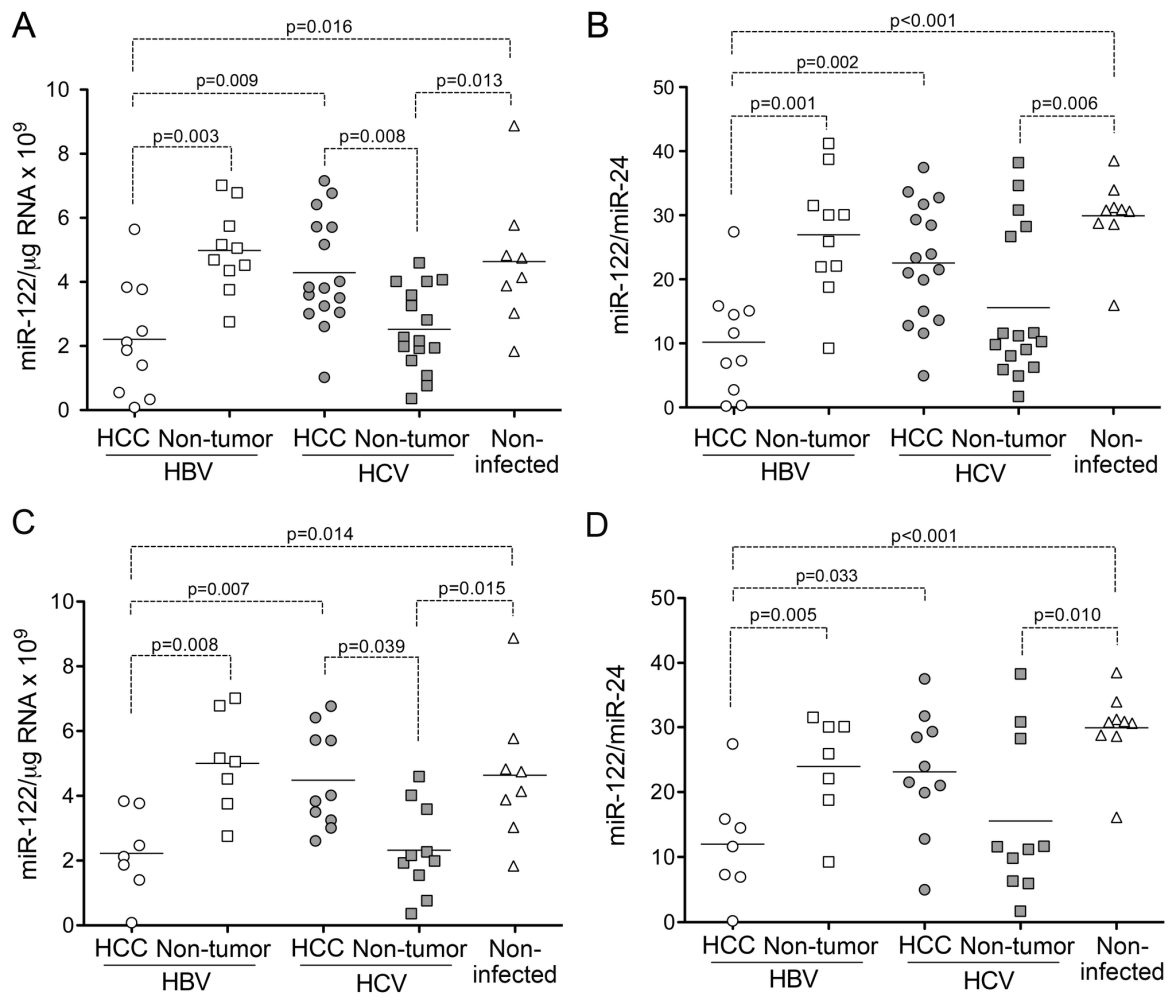
miR-122 expression in paired HCC and non-tumor liver tissue from patients with chronic HBV and HCV infection and control, non-infected liver tissue. (**A**) miR-122 abundance quantified by qRT-PCR in paired tumor and non-tumor tissues and non-infected (‘normal’) liver from patients undergoing resection of metastatic tumors, normalized to total RNA. (**B**) Relative miR-122 abundance normalized to miR-24 abundance in the same tissues. (**C**) miR-122 abundance in HCC classified histologically as “moderately differentiated”, paired non-tumor tissue from the same patients, and non-infected (‘normal’) liver. (**D**) miR-122 abundance in the subset of tissues shown in panel C, normalized to miR-24 abundance. The statistical significance of differences between paired observations was estimated using the paired t test, while differences between non-paired observations were analyzed by the Mann-Whitney test.

qRT-PCR revealed significant differences in the abundance of miR-122 in both tumor and non-tumor tissue samples when the HBV- and HCV-infected groups were compared (Figure 2). miR-122 abundance (miR-122 copy number per µg total RNA) was significantly lower in HCC tissue from HBV-infected versus HCV-infected subjects (p=0.009 by two-sided t test). In contrast, the miR-122 abundance in non-tumor tissue from HBV-infected patients was significantly greater than that in the HCV-infected patients (p=0.0005 by two-sided t test). The mean miR-122 abundance in HCC tissue was less than half that in non-tumor tissue in HBV-infected patients (p=0.003 by two-sided, paired t test). Strikingly, this relationship was reversed in the HCV-infected patients, in whom miR-122 abundance in HCC tissue was almost twice that in the non-tumor tissue (p=0.008 by two-sided paired t test). There was no significant difference in the abundance of miR-122 in the non-tumor tissue from HBV-infected patients and HCV-associated HCC. miR-122 abundance varied quite widely in liver tissue collected from non-infected individuals undergoing resection of metastatic tumors. Despite this, miR-122 abundance was significantly less in HBV-associated cancer tissue and non-tumor HCV-infected tissue than in the non-infected tissues (p=0.016 and 0.013, respectively).

**Figure 3:**
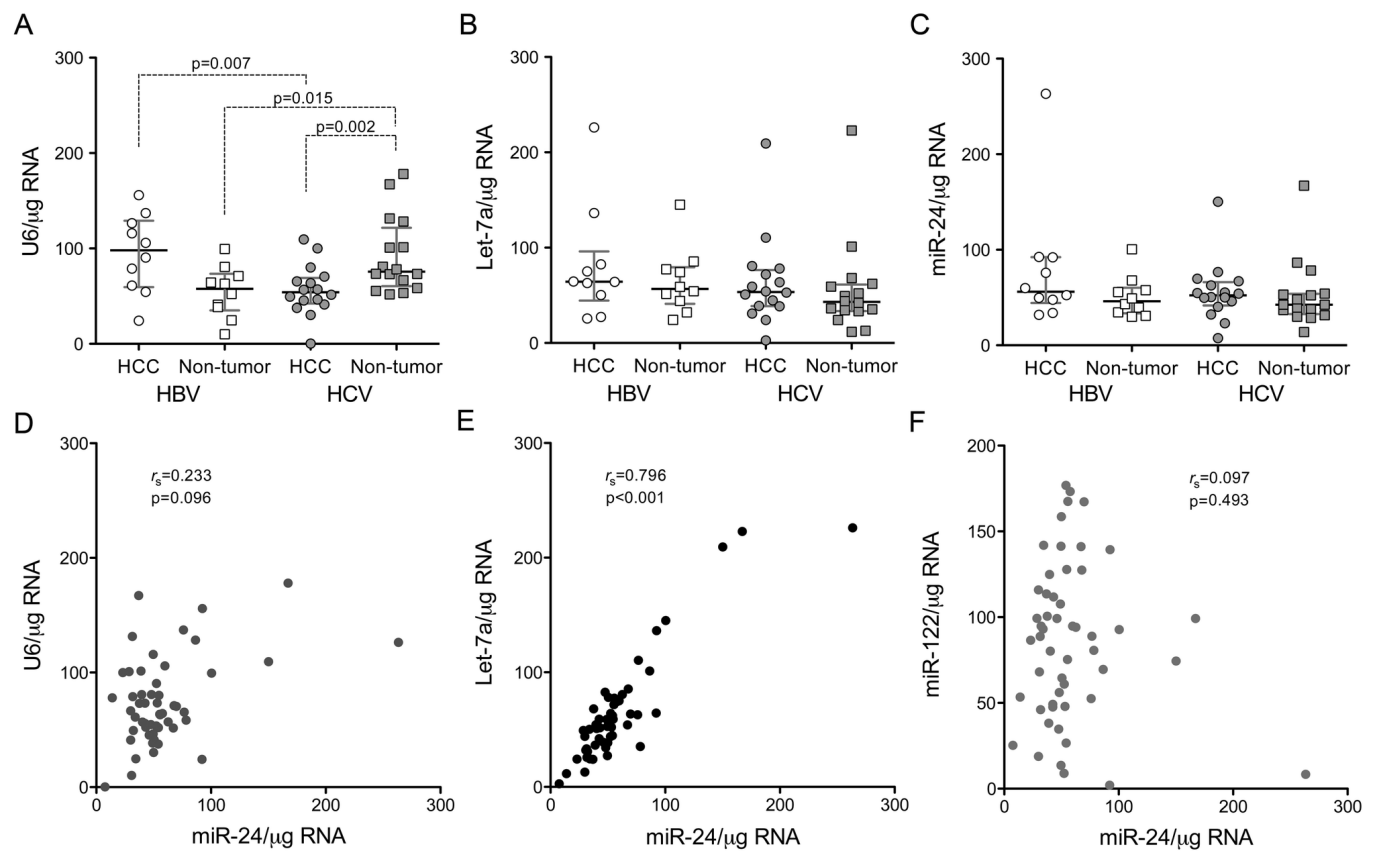
Comparison of small RNAs as normalizers for assessing miR-122 abundance. Shown in the panels at the top are the relative abundance of (**A**) U6 snRNA, (**B**) Let-7a, and (**C**) miR-24 miRNAs in paired tumor and non-tumor tissues from subjects with HBV or HCV infection, normalized to total RNA. Bars represent median and quartiles for each group. Statistical comparisons between groups were made with paired or unpaired t tests, and are shown only if p<.05. In the lower set of panels, (**D**) U6, (**E**) Let-7a, and (**F**) miR-122 abundance are plotted as a function of miR-24 abundance. r_s_ = Spearman rank-order correlation coefficient.

To account for potential differences in degradation of the RNA or efficiency of reverse transcription between tissue samples, we assessed the abundance of several other small RNAs against which we could normalize the abundance of miR-122. U6, a noncoding snRNA component of the spliceosome, is commonly used to normalize miRNA abundance. However, we observed substantial differences in U6 abundance in these tissues, suggesting that U6 would be a poor normalizer (Figure 3A). Substantially less variation was observed in the abundance of the miRNAs, miR-24 or Let-7a (Figure 3B and C), for which the standard deviation of the critical threshold [25] in the PCR assay was 0.79 and 1.27, respectively, compared to 1.34 for U6. Notably, we observed no difference in the abundance of Let-7a in HBV-associated cancer and non-tumor tissues (p=0.52 by two-sided, paired t test), despite a prior report suggesting that Let-7a expression is regulated by the HBx protein and increased in abundance in HBV-associated HCC [32]. In addition, although miR-24 negatively regulates the expression of hepatocyte nuclear factor 4-alpha (HNF4-alpha) and thus might be up-regulated in some liver cancers [33], we did not observe this. A strong positive correlation was evident between the abundance of miR-24 and Let-7a (Figure 3E, Spearman r_s_=0.7959, p<0.001 by two-tailed t test), suggesting that these miRNAs might belong to a common regulatory network and that either could be used to normalize miR-122 abundance. In contrast, there was no correlation between miR-24 and either U6 or miR-122 abundance (Figure 3D and F), which indicates that U6 and miR-122 are regulated independently of miR-24. Importantly, when the miR-122 abundance was normalized to miR-24 levels, miR-122 expression remained significantly depressed in HBV-associated HCC when compared either with paired non-tumor tissue, or HCC tissue from HCV-infected subjects (p<0.001 and p=0.002, respectively, Figure 2B). In replicate assays, the abundance of miR-122 in non-tumor HCV-infected tissue also remained significantly lower than either non-infected or HBV-infected liver tissues (Figure 2B). Similar associations were found when miR-122 abundance was normalized to Let-7a (data not shown).

To assess further the possibility of bias in these results due to differences in the quality of the RNA samples, we compared the RNA integrity number (RIN) [29] of each sample with the abundance of each of the small RNAs detected. Interestingly, while the quantity of U6 snRNA detected correlated positively with the RIN score (Spearman r_*s*_ = 0.5216, two-tailed p = 0.0001) (Figure S1A in Supporting Information), this was not the case with miR-24 or Let-7a (r_*s*_ = -0.124 and -0.045, respectively). RIN scores also did not vary significantly between tumor and non-tumor tissue-derived RNA samples, or RNA from HBV- vs. HCV-infected tissue. Thus, although the quality of the RNA samples was generally high (mean RIN = 8.0 ± 0.17 s.e.m.), it was an important factor in determining the abundance of U6 but not either of these miRNAs. These data suggest that U6 is less stable than the miRNAs and provide additional support for the use of miR-24 (or Let-7a) as a standard against which to normalize miR-122 abundance (see Discussion). Nonetheless, when miR-122 results were normalized to U6 abundance, the correlations described above between miR-122 abundance, in both tumor and non-tumor tissues, and the type of virus infection remained strongly statistically significant (Figure S1B in Supporting Information). The mean miR-122 abundance was substantially lower in HBV-associated HCC tissue than in HBV-infected non-tumor tissue (p = 0.003 by paired t-test), while this relationship was reversed in HCV-infected liver (p = 0.001). miR-122 abundance was also significantly lower in non-tumor tissue from HCV-infected subjects than HBV-infected subjects (p < 0.001).

To exclude the possibility of bias due to the trend toward a less differentiated histologic classification among HBV-associated cancers (Figure 1B), we limited the comparison of miR-122 abundance to those HCC tissues that were scored as moderately differentiated and their corresponding paired non-tumor samples. While this reduced the number of subjects available for analysis, miR-122 abundance remained significantly lower in HBV- versus HCV-associated cancer tissue: p=0.007 when compared on the basis of miR-122 copy number/mg RNA (Figure 2C) vs. p=0.033 when normalized to miR-24 (Figure 2D). Thus differences in miR-122 abundance in HCC associated with HBV vs. HCV infection are independent of the degree of histologic differentiation of the cancer.

Collectively, these results provide strong evidence that miR-122 expression is reduced in HCC associated with HBV infection, but not in most HCV-associated liver cancers.

### Reduced miR-122 abundance is associated with interferon responses in HCV-infected liver

**Figure 4:**
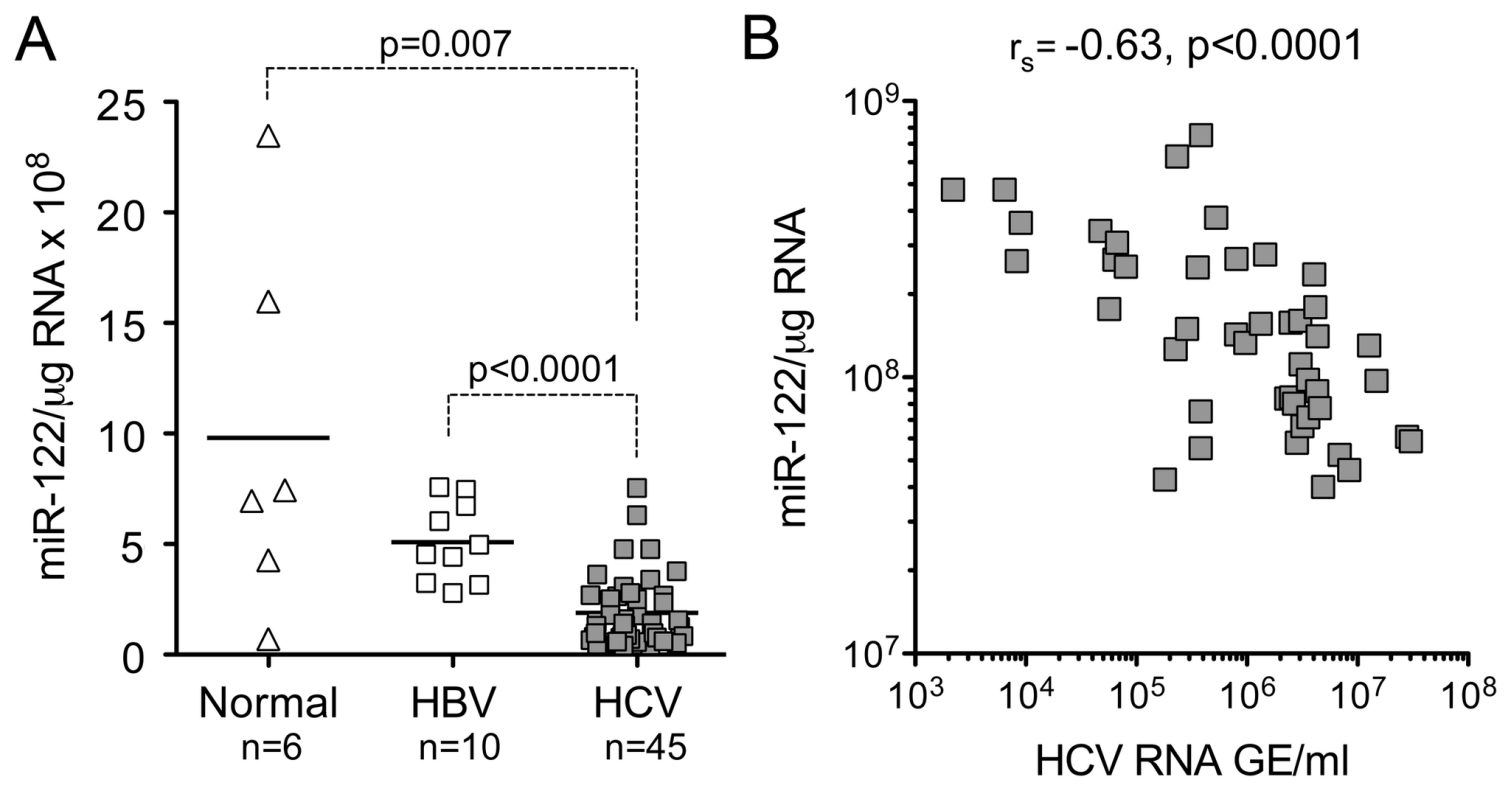
miR-122 expression in chimpanzee liver tissue. (**A**) Hepatic miR-122 abundance in liver biopsies from chimpanzees infected with HBV or HCV, or not infected with either virus (‘normal’). Statistical significance was assessed by non-paired two-sided t test. Bars represent mean values. (**B**) Liver miR-122 expression plotted against serum HCV RNA abundance from acutely HCV-infected chimpanzees. r_s_ = Spearman rank-order correlation coefficient.

The data shown in Figure 2 indicate that miR-122 is frequently reduced in abundance in non-tumor, HCV-infected liver tissue, but not in liver infected with HBV. To determine whether similar HCV-induced suppression of miR-122 expression occurs in chimpanzees (*Pan troglodytes*), the only animal species other than humans that is permissive for HCV infection, we measured miR-122 abundance in liver tissues collected previously from 45 HCV-infected chimpanzees, and compared this to that present in 10 HBV-infected animals, and 6 that were not infected with either virus. These results showed that miR-122 expression was significantly reduced in HCV-infected liver compared to both HBV-infected (p<0.0001) and normal, non-infected (p=0.007) chimpanzee liver (Figure 4A). A strong, negative correlation (Spearman r_s_= -0.63, p<0.0001) existed between hepatic miR-122 expression levels and HCV RNA copy numbers in serum (Figure 4B). The mean miR-122 abundance was lower in HBV-infected liver than in uninfected chimpanzee liver (Figure 4A), but the difference did not achieve statistical significance (p=0.059 by two-tailed t test). Thus, intra-hepatic miR-122 abundance is reduced in HCV-infected chimpanzees as well as humans. This is consistent with earlier studies that have found reduced intrahepatic expression of miR-122 in patients with advanced chronic hepatitis C [34–36].

**Figure 5:**
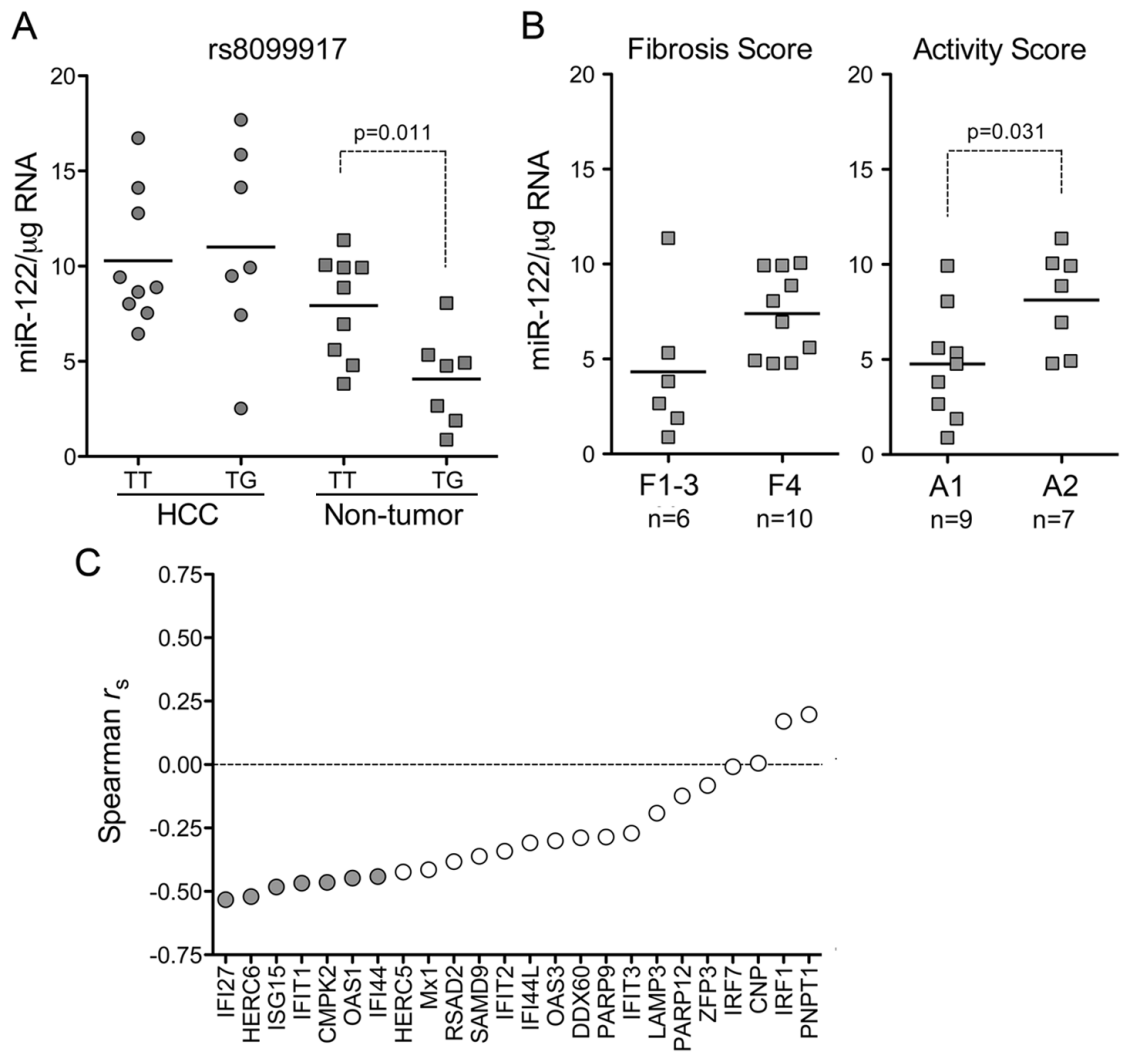
miR-122 expression, IL28B genotype, Metavir scores and ISG transcript levels in HCV-infected human liver. (**A**) miR-122 expression in HCC and paired non-tumor samples from subjects with HCV infection, grouped according to rs8099917 genotype (TT or GT). (**B**) miR-122 expression levels in non-tumor tissue from HCV-infected subjects categorized according to Metavir score for (left) fibrosis and (right) inflammatory activity. (**C**) Correlation between miR-122 abundance and expression levels of selected ISGs determined by Affymetrix U133 Plus 2.0 Array analysis. With the exception of OAS1 and Mx1, intrahepatic transcript levels of these ISGs have been shown previously to be predictive of Peg-IFN/RBV treatment outcome [31]. “r_s_” = Spearman rank-order correlation coefficient. Filled symbols indicate a statistically significant negative correlation (p<0.05 by one-sided t test).

Sarasin-Filipowicz et al. [36] reported previously that miR-122 levels were reduced in liver from HCV-infected patients who responded poorly to treatment with pegylated IFN-α and ribavirin (Peg-IFN/RBV). Consistent with this, we observed a negative correlation between miR-122 abundance in non-tumor tissue from HCV-infected human subjects and the GT versus TT genotype at the rs8099917 locus in the IL28B gene (p=0.011, Figure 5A) that is predictive of a poor response to Peg-IFN/RBV therapy [37]. HCV-infected patients with the TT genotype are prone to a greater inflammatory response than those with TG or GG [38]. Thus, differences in IL28B genotype may have contributed to a correlation we observed between miR-122 abundance and A1 versus A2 Metavir activity scores (Figure 5B, 6 of 7 subjects with an A2 Metavir score had the TT genotype). Importantly, the association between IL28B genotype and miR-122 abundance was observed only in non-tumor liver from HCV-infected patients, and not in paired HCC tissue (Figure 5A).

Patients who are non-responsive to Peg-IFN/RBV, or who have IL28B genotypes predictive of a poor response to Peg-IFN/RBV therapy, are likely to have increased pre-treatment intra-hepatic ISG transcript levels compared to those who respond well to treatment [39–41]. We thus asked whether a correlation existed between miR-122 abundance and levels of selected ISG transcripts in HCV-infected non-tumor tissue determined by Affymetrix 133U Plus 2.0 GeneChip assay. For this analysis, we selected ISGs that were shown previously to be correlated with treatment response [39] (Figure 5C). We also included Mx1 and OAS1, both well-characterized ISGs. Overall, the Affymetrix signals for these genes showed a strong trend toward negative correlations with miR-122 abundance. Fourteen of 24 ISGs demonstrated a Spearman rank-order coefficient, r_s_, ≤ -0.300; this negative correlation was significant (p<0.05) for 7 of the ISGs by one-tailed t test (Figure 3C). These data are consistent with the notion that reduced miR-122 abundance is associated with strong intrahepatic IFN-mediated responses to the virus.

### miR-191 abundance is increased in HBV-associated HCC

**Figure 6:**
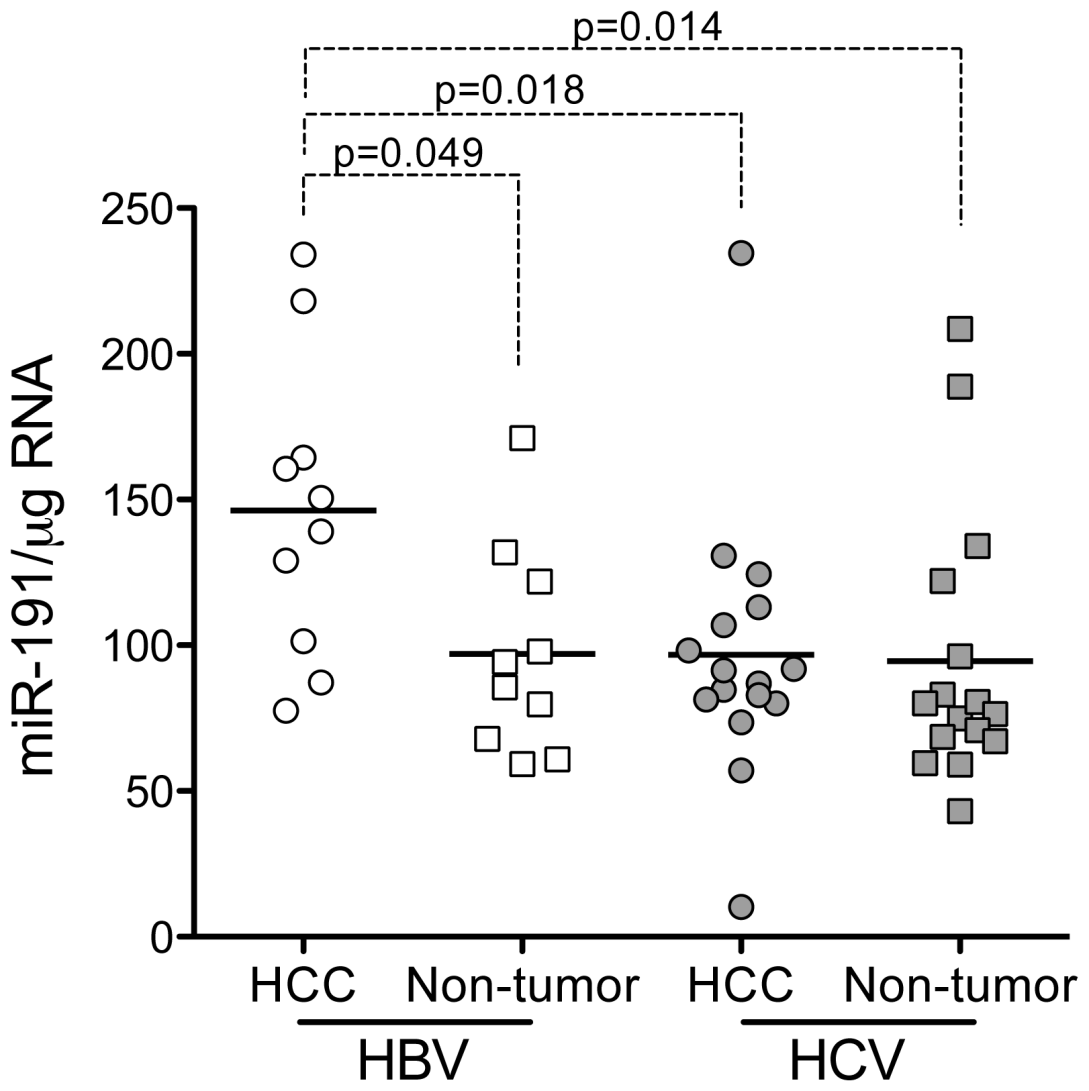
Relative abundance of miR-191 in paired HCC and non-tumor tissue from subjects with HBV or HCV infection. Relative miR-191 abundance in paired tumor and non-tumor samples from HBV- and HCV-infected subjects normalized to total RNA. Statistical significance was assessed in two-sided paired t tests for comparisons between tumor and non-tumor tissue, or two-sided unpaired t test for comparison between infection groups.

Since Elyakim et al. [42] reported recently that miR-191 was increased in HCC arising in a study population comprised mostly of HBV-infected subjects, we also quantified miR-191 expression levels in the human tissue samples. We confirmed miR-191 levels were modestly increased in HBV-associated HCC compared to non-tumor HBV-infected tissue when normalized to total RNA (p=0.049 by two-sided, paired t test, Figure 6). This trend remained significant only by one-sided t test when the miR-191 abundance was normalized to miR-24 abundance (p=0.045), and was absent when miR-191 levels were normalized to U6 snRNA. miR-191 abundance in non-tumor, HBV-infected tissue was similar to that in both tumor and non-tumor liver from HCV-infected subjects (Figure 6).

## Discussion/Conclusions

miR-122 is a critical regulator of hepatic gene expression and an essential host factor for HCV replication [15,18,43]. It also has important tumor suppressor properties [16,44], and recent reports indicate that loss of its expression promotes carcinogenesis in knockout mice [45,46]. While its abundance is often reduced in HCC [21,22], two previous studies suggest that miR-122 expression may be preserved in liver cancer arising in patients with HCV infection [24,25]. We confirm this, showing in a genetically and geographically homogeneous population of patients, and normalizing results either to total RNA or to levels of miR-24, that miR-122 abundance is significantly reduced from normal in HBV-associated HCC but not in liver cancer associated with HCV infection (Figure 2A and B). This difference in miR-122 expression is independent of the histologic classification of the tumors (Figure 2C and D), as well as the degree of fibrosis or inflammation in paired non-tumor tissue from the same patients. Conversely, we show that miR-191 tends to be increased in abundance in HBV-associated cancer, but not HCV-associated HCC (Figure 6). These observations have important implications for the pathogenetic mechanisms involved in viral carcinogenesis within the liver. While HCC may arise as a result of factors common to both HBV and HCV infection (such as chronic inflammation, oxidative stress, and progressive fibrosis leading to cirrhosis), distinctive molecular signatures associated with HBV- versus HCV-associated cancer suggest there are fundamental differences in the ways these two viruses cause cancer.

Our study highlights the challenges inherent in comparing miRNA abundance in different clinical samples. In addition to potential differences in the proportion of cells present within a biopsy that are of hepatocellular origin vs. derived from other cell lineages, a constant concern is the quality of the RNA. While our initial analysis, like many studies, compared miR-122 copy numbers based on the quantity of total RNA subjected to RT-PCR, this approach can be biased by differences in the quality of the RNA and degree of RNA degradation. Although our RNA samples were of generally high quality (see Figure S1A in Supporting Information), we determined miR-24, Let-7a, and U6 snRNA copy numbers and evaluated each as a standard against which miR-122 abundance could be normalized to account for potential differences in RNA integrity (Figure 3). Median miR-24 and Let-7a copy numbers did not vary significantly between tumor and non-tumor tissue samples from HBV- and HCV-infected subjects (one-way ANOVA), suggesting that the expression of these miRNAs is relatively constant in liver and that either could serve as a standard for normalizing miR-122 abundance. In contrast, median U6 copy numbers varied significantly between these tissue groups (p = 0.004 by one-way ANOVA with Kruskal-Wallis test) and, more importantly, were strongly correlated negatively with the RIN score, a measure of RNA integrity [29] (Figure S1A in Supporting Information). There was no correlation between the RIN score and miR-24 or Let-7a abundance, suggesting that U6 snRNA may be less stable and more prone to degradation than the miRNAs. This may be due to the greater length of U6 (106 nts vs. ~20-23 nts for miRNAs), or the absence of terminal modifications that may influence the stability of miRNAs [47]. Consistent with this, Let-7a was found to have greater biological stability and to be superior to U6 for normalization of miRNA abundance in previous studies of rat hepatocyte RNA [48]. Nonetheless, even though these data argue against the use of U6 as a standard for normalizing miR-122 copy numbers, we found the abundance of miR-122 was significantly reduced in HCC associated with HBV but not HCV infection, and that miR-122 abundance was significantly depressed in non-tumor tissue infected with HCV but not HBV, using any of these small RNAs, including U6, to normalize the miR-122 results.

While it remains unclear exactly how miR-122 contributes to the HCV lifecycle, it is known to promote viral replication independently of its regulation of hepatic genes [49]. It binds to two sites near the 5’ end of the viral genome [18], recruiting argonaute 2 (EIF2C2) and physically stabilizing the RNA by protecting it from 5’ exonucleolytic Xrn1-mediated decay [19,20]. However, miR-122 has other, positive effects on HCV replication beyond its ability to physically stabilize the viral genome [20,50]. It is essential for HCV replication, and its therapeutic silencing with an antisense oligonucleotide has potent antiviral effects [15,51]. No other RNA virus is known to rely so completely on a cellular miRNA for its replication cycle. Thus, the continued expression of miR-122 in HCV-associated HCC could reflect close linkage between carcinogenesis and HCV replication and viral protein expression. We speculate that miR-122 expression is preserved in HCV-associated HCC (in contrast to HBV-associated cancer) because HCV-encoded proteins help to drive a multi-stage process of carcinogenesis within infected cells. This may result from the ability of the virus to directly disable DNA damage responses or other cellular tumor suppressor functions, and to contribute directly to malignant conversion of hepatocytes as reviewed elsewhere [10,52]. Early loss of miR-122 during the progression to cancer would eliminate virus replication, protecting the cell from further effects of viral protein expression. In contrast, in HBV-infected cells, a loss of miR-122 expression could both accelerate tumorigenesis and enhance replication, as miR-122 appears to restrict, rather than promote, HBV replication [53–56]. Although speculative, this hypothesis raises the interesting possibility that HCV-associated cancers arise within the small minority of hepatocytes infected with the virus, and not the much larger number of uninfected bystander cells [52,57].

Epigenetic mechanisms are likely to contribute to the differential expression of miR-122 and miR-191 in HCC. The miR-122 promoter is hyper-methylated in the HBV-associated HCC-derived cell line, Hep3B [58]. It remains to be seen whether differences exist in methylation of the promoter in vivo in HBV- versus HCV-associated cancers, but bacterial artificial chromosome array-based methylated CpG island amplification (BAMCA) studies indicate significant differences in the methylation patterns present in HBV- and HCV-associated HCC [59]. The HBx protein expressed by HBV may influence cellular methyltransferase activity, and could possibly contribute to altered methylation patterns [60]. An additional possibility is that epigenetic differences in HBV- and HCV-associated HCC could reflect different cell types from which the cancer originates, as HBV may be capable of infecting hepatocyte progenitors [61].

Our results also show that miR-122 expression is reduced in non-tumor liver tissue from HCV-infected persons. In contrast, contrary to a recent report by Wang et al. [54], we found that miR-122 is expressed at normal levels in non-tumor HBV-infected liver (Figure 2). Several lines of evidence suggest that this difference may reflect a more active IFN response in HCV- versus HBV- infected livers. In vitro studies suggest that IFN-β inhibits miR-122 expression [36,62], and HCV stimulates a more robust intrahepatic innate immune response than HBV [63,64]. Consistent with this, our results reveal a correlation between miR-122 abundance in non-tumor tissues and IL28B genotype, defined by a single nucleotide polymorphism (rs8099917) associated with response to Peg-IFN/RBV as well as endogenous pre-treatment ISG expression levels (Figure 5A) [40,41]. We also found an inverse relationship between the abundance of several ISG transcripts and miR-122 (Figure 5C). Interestingly, this relationship was not observed in tumor tissues from these patients, suggesting that genetic or epigenetic changes alter miR-122 regulation in HCC tissue, or that the cancer cells are refractory to stimulation by type 1 IFNs.

Consistent with our findings in HCV-infected patients, we also observed a reduction in miR-122 abundance in liver tissue from HCV-infected chimpanzees (Figure 4A), and an inverse correlation between the abundance of HCV RNA in the liver and serum HCV RNA levels (Figure 4B). Although Sarasin-Filopowicz et al. [36] demonstrated a trend toward lower miR-122 abundance in liver tissues with high viral RNA copy numbers, this did not achieve statistical significance and no correlation was evident between serum RNA levels and miR-122 abundance in the patients studied by this group. It is not clear why such a relationship exists in chimpanzees but not infected humans. One possibility is that it might be related to the fact that chimpanzees generally have very robust intrahepatic innate immune responses to HCV, with uniformly high levels of intrahepatic ISG expression [65]. The uniformly high intrahepatic innate immune response in chimpanzees contrasts with extensive variation in the intensity of ISG responses in HCV-infected humans [39], possibly allowing for a negative correlation between serum viral RNA level and miR-122 abundance to become manifest.

Finally, our results indicate that miR-191 expression may be increased in HBV-associated HCC (Figure 6). This supports a previous study in which miR-191 abundance was increased in HCC of mixed origin, but predominantly associated with HBV infection [42]. miR-191 antagonism has been shown to have anti-tumor potential in studies of Hep3B and SNU423 cells [42], which are both derived from HBV-associated cancers. Our data suggest that elevations of miR-191 are confined to HBV-associated liver cancer (Figure 6), and suggest that virus-specific differences in miRNA signatures may be important in understanding the origins of liver cancer. While these differences may be predictive of response to specific therapeutic interventions, they are unlikely to be of sufficient magnitude or specificity to guide therapy in individual patients.

## Supporting Information

Figure S1 **U6 snRNA copy number as a standard for normalization of miR-122 abundance.** (**A**) U6 copy number (relative copy number per µg RNA) plotted as a function of the RNA integrity number (RIN score, on a scale of 1 to 10) determined as described in Methods in the main text. A strong negative correlation exists between U6 copy number and the RIN score: Spearman r_*s*_ = 0.5216, two-tailed p = 0.0001). (**B**) miR-122 abundance in HCC and non-tumor tissues from HBV- and HCV-infected subjects, normalized to U6 snRNA copy number. Statistical significance was assessed using paired and unpaired t tests, as described in the main text.
(TIF)

## References

[1] Ferlay JSH, Bray F, Forman D, Mathers C, Parkin DM (2010). ancer Incidence Mortal Worldwide IARC CancerBase No. 10

[2] Wang XGJ, Thorgeirsson S, editors (2011) Molecular Genetics of Liver Neoplasia. New York: Springer Verlag Science pp. 51-73.

[3] Lok AS, Everhart JE, Wright EC, Di Bisceglie AM, Kim HY et al. (2011) Maintenance peginterferon therapy and other factors associated with hepatocellular carcinoma in patients with advanced hepatitis C. Gastroenterology 140: 840-849. doi:10.1053/j.gastro.2010.11.050. PubMed: 21129375. 21129375 PMC3057272

[4] White D, El-Serag H (2011) Epidemiology of Hepatocellular Carcinoma. In: Wang X [!(surname)!] Thorgeirsson S Molecular Genetics of Liver Neoplasia. New York, NY: Springer Verlag Science pp. 51-73.

[5] Kiyosawa K, Umemura T, Ichijo T, Matsumoto A, Yoshizawa K et al. (2004) Hepatocellular carcinoma: recent trends in Japan. Gastroenterology 127: S17-S26. doi:10.1053/j.gastro.2004.03.068. PubMed: 15508082. 15508082

[6] McGivern DR, Lemon SM (2011) Virus-specific mechanisms of carcinogenesis in hepatitis C virus associated liver cancer. Oncogene 30: 1969-1983. doi:10.1038/onc.2010.594. PubMed: 21258404. 21258404 PMC3642622

[7] Tsai WL, Chung RT (2010) Viral hepatocarcinogenesis. Oncogene 29: 2309-2324. doi:10.1038/onc.2010.36. PubMed: 20228847. 20228847 PMC3148694

[8] Nakamoto Y, Guidotti LG, Kuhlen CV, Fowler P, Chisari FV (1998) Immune pathogenesis of hepatocellular carcinoma. J Exp Med 188: 341-350. doi:10.1084/jem.188.2.341. PubMed: 9670046. 9670046 PMC2212453

[9] Lerat H, Honda M, Beard MR, Loesch K, Sun J et al. (2002) Steatosis and liver cancer in transgenic mice expressing the structural and nonstructural proteins of hepatitis C virus. Gastroenterology 122: 352-365. doi:10.1053/gast.2002.31001. PubMed: 11832450. 11832450

[10] McGivern DR, Lemon SM (2009) Tumor suppressors, chromosomal instability, and hepatitis C virus-associated liver cancer. Annu Rev Pathol 4: 399-415. doi:10.1146/annurev.pathol.4.110807.092202. PubMed: 18928409. 18928409 PMC4422400

[11] Wilson RC, Doudna JA (2013) Molecular mechanisms of RNA interference. Annu Rev Biophys 42: 217-239. doi:10.1146/annurev-biophys-083012-130404. PubMed: 23654304. 23654304 PMC5895182

[12] Djuranovic S, Nahvi A, Green R (2011) A parsimonious model for gene regulation by miRNAs. Science 331: 550-553. doi:10.1126/science.1191138. PubMed: 21292970. 21292970 PMC3955125

[13] Meijer HA, Kong YW, Lu WT, Wilczynska A, Spriggs RV et al. (2013) Translational repression and eIF4A2 activity are critical for microRNA-mediated gene regulation. Science 340: 82-85. doi:10.1126/science.1231197. PubMed: 23559250. 23559250

[14] Chang J, Nicolas E, Marks D, Sander C, Lerro A et al. (2004) miR-122, a mammalian liver-specific microRNA, is processed from hcr mRNA and may downregulate the high affinity cationic amino acid transporter CAT-1. RNA Biol 1: 106-113. doi:10.4161/rna.1.2.1066. PubMed: 17179747. 17179747

[15] Lanford RE, Hildebrandt-Eriksen ES, Petri A, Persson R, Lindow M et al. (2010) Therapeutic silencing of microRNA-122 in primates with chronic hepatitis C virus infection. Science 327: 198-201. doi:10.1126/science.1178178. PubMed: 19965718. 19965718 PMC3436126

[16] Bai S, Nasser MW, Wang B, Hsu SH, Datta J et al. (2009) MicroRNA-122 inhibits tumorigenic properties of hepatocellular carcinoma cells and sensitizes these cells to sorafenib. J Biol Chem 284: 32015-32027. doi:10.1074/jbc.M109.016774. PubMed: 19726678. 19726678 PMC2797273

[17] Lewis AP, Jopling CL (2010) Regulation and biological function of the liver-specific miR-122. Biochem Soc Trans 38: 1553-1557. doi:10.1042/BST0381553. PubMed: 21118125. 21118125

[18] Jopling CL, Yi M, Lancaster AM, Lemon SM, Sarnow P (2005) Modulation of hepatitis C virus RNA abundance by a liver-specific MicroRNA. Science 309: 1577-1581. doi:10.1126/science.1113329. PubMed: 16141076. 16141076

[19] Shimakami T, Yamane D, Jangra RK, Kempf BJ, Spaniel C et al. (2012) Stabilization of hepatitis C virus RNA by an Ago2-miR-122 complex. Proc Natl Acad Sci U S A 109: 941-946. doi:10.1073/pnas.1112263109. PubMed: 22215596. 22215596 PMC3271899

[20] Li Y, Masaki T, Yamane D, McGivern DR, Lemon SM (2013) Competing and noncompeting activities of miR-122 and the 5' exonuclease Xrn1 in regulation of hepatitis C virus replication. Proc Natl Acad Sci U S A 110: 1881-1886. doi:10.1073/pnas.1213515110. PubMed: 23248316. 23248316 PMC3562843

[21] Kutay H, Bai S, Datta J, Motiwala T, Pogribny I et al. (2006) Downregulation of miR-122 in the rodent and human hepatocellular carcinomas. J Cell Biochem 99: 671-678. doi:10.1002/jcb.20982. PubMed: 16924677. 16924677 PMC3033198

[22] Gramantieri L, Ferracin M, Fornari F, Veronese A, Sabbioni S et al. (2007) Cyclin G1 is a target of miR-122a, a microRNA frequently down-regulated in human hepatocellular carcinoma. Cancer Res 67: 6092-6099. doi:10.1158/0008-5472.CAN-06-4607. PubMed: 17616664. 17616664

[23] Hou J, Lin L, Zhou W, Wang Z, Ding G et al. (2011) Identification of miRNomes in human liver and hepatocellular carcinoma reveals miR-199a/b-3p as therapeutic target for hepatocellular carcinoma. Cancer Cell 19: 232-243. doi:10.1016/j.ccr.2011.01.001. PubMed: 21316602. 21316602

[24] Varnholt H, Drebber U, Schulze F, Wedemeyer I, Schirmacher P et al. (2008) MicroRNA gene expression profile of hepatitis C virus-associated hepatocellular carcinoma. Hepatology 47: 1223-1232. PubMed: 18307259. 18307259 10.1002/hep.22158

[25] Coulouarn C, Factor VM, Andersen JB, Durkin ME, Thorgeirsson SS (2009) Loss of miR-122 expression in liver cancer correlates with suppression of the hepatic phenotype and gain of metastatic properties. Oncogene 28: 3526-3536. doi:10.1038/onc.2009.211. PubMed: 19617899. 19617899 PMC3492882

[26] Bedossa P, Poynard T (1996) An algorithm for the grading of activity in chronic hepatitis C. The METAVIR Cooperative Study Group. Hepatology 24: 289-293. doi:10.1002/hep.510240201. PubMed: 8690394. 8690394

[27] Desmet VJ, Gerber M, Hoofnagle JH, Manns M, Scheuer PJ (1994) Classification of Chronic Hepatitis: Diagnosis, Grading and Staging. Hepatology 19: 1513-1520. doi:10.1002/hep.1840190629. PubMed: 8188183. 8188183

[28] Honda M, Sakai A, Yamashita T, Nakamoto Y, Mizukoshi E et al. (2010) Hepatic ISG expression is associated with genetic variation in interleukin 28B and the outcome of IFN therapy for chronic hepatitis C. Gastroenterology 139: 499-509. doi:10.1053/j.gastro.2010.04.049. PubMed: 20434452. 20434452

[29] Schroeder A, Mueller O, Stocker S, Salowsky R, Leiber M et al. (2006) The RIN: an RNA integrity number for assigning integrity values to RNA measurements. BMC Mol Biol 7: 3. doi:10.1186/1471-2199-7-3. PubMed: 16448564. 16448564 PMC1413964

[30] Lanford RE, Guerra B, Lee H, Averett DR, Pfeiffer B et al. (2003) Antiviral effect and virus-host interactions in response to alpha interferon, gamma interferon, poly(i)-poly(c), tumor necrosis factor alpha, and ribavirin in hepatitis C virus subgenomic replicons. J Virol 77: 1092-1104. doi:10.1128/JVI.77.2.1092-1104.2003. PubMed: 12502825. 12502825 PMC140845

[31] Nagaoki Y, Hyogo H, Aikata H, Tanaka M, Naeshiro N et al. (2012) Recent trend of clinical features in patients with hepatocellular carcinoma. Hepatol Res 42: 368-375. doi:10.1111/j.1872-034X.2011.00929.x. PubMed: 22151896. 22151896

[32] Wang Y, Lu Y, Toh ST, Sung WK, Tan P et al. (2010) Lethal-7 is down-regulated by the hepatitis B virus x protein and targets signal transducer and activator of transcription 3. J Hepatol 53: 57-66. doi:10.1016/j.jhep.2009.12.043. PubMed: 20447714. 20447714

[33] Hatziapostolou M, Polytarchou C, Aggelidou E, Drakaki A, Poultsides GA et al. (2011) An HNF4alpha-miRNA inflammatory feedback circuit regulates hepatocellular oncogenesis. Cell 147: 1233-1247. doi:10.1016/j.cell.2011.10.043. PubMed: 22153071. 22153071 PMC3251960

[34] Trebicka J, Anadol E, Elfimova N, Strack I, Roggendorf M et al. (2013) Hepatic and serum levels of miR-122 after chronic HCV-induced fibrosis. J Hepatol 58: 234-239. doi:10.1016/S0168-8278(13)60572-3. PubMed: 23085648. 23085648

[35] Morita K, Taketomi A, Shirabe K, Umeda K, Kayashima H et al. (2011) Clinical significance and potential of hepatic microRNA-122 expression in hepatitis C. Liver Int 31: 474-484. doi:10.1111/j.1478-3231.2010.02433.x. PubMed: 21199296. 21199296

[36] Sarasin-Filipowicz M, Krol J, Markiewicz I, Heim MH, Filipowicz W (2009) Decreased levels of microRNA miR-122 in individuals with hepatitis C responding poorly to interferon therapy. Nat Med 15: 31-33. doi:10.1038/nm.1902. PubMed: 19122656. 19122656

[37] Tanaka Y, Nishida N, Sugiyama M, Kurosaki M, Matsuura K et al. (2009) Genome-wide association of IL28B with response to pegylated interferon-alpha and ribavirin therapy for chronic hepatitis C. Nat Genet 41: 1105-1109. doi:10.1038/ng.449. PubMed: 19749757. 19749757

[38] Yoshizawa H (2002) Hepatocellular carcinoma associated with hepatitis C virus infection in Japan: projection to other countries in the foreseeable future. Oncology 62 Suppl 1: 8-17. doi:10.1159/000048270. PubMed: 11868791. 11868791

[39] Sarasin-Filipowicz M, Oakeley EJ, Duong FH, Christen V, Terracciano L et al. (2008) Interferon signaling and treatment outcome in chronic hepatitis C. Proc Natl Acad Sci U S A 105: 7034-7039. doi:10.1073/pnas.0707882105. PubMed: 18467494. 18467494 PMC2383932

[40] Urban TJ, Thompson AJ, Bradrick SS, Fellay J, Schuppan D et al. (2010) IL28B genotype is associated with differential expression of intrahepatic interferon-stimulated genes in patients with chronic hepatitis C. Hepatology 52: 1888-1896. doi:10.1002/hep.23912. PubMed: 20931559. 20931559 PMC3653303

[41] Abe H, Hayes CN, Ochi H, Maekawa T, Tsuge M et al. (2011) IL28 variation affects expression of interferon stimulated genes and peg-interferon and ribavirin therapy. J Hepatol 54: 1094-1101. doi:10.1002/hep.24499. PubMed: 21145800. 21145800

[42] Elyakim E, Sitbon E, Faerman A, Tabak S, Montia E et al. (2010) hsa-miR-191 is a candidate oncogene target for hepatocellular carcinoma therapy. Cancer Res 70: 8077-8087. doi:10.1158/0008-5472.CAN-10-1313. PubMed: 20924108. 20924108

[43] Jangra RK, Yi M, Lemon SM (2010) miR-122 regulation of hepatitis C virus translation and infectious virus production. J Virol 84: 6615-6625. doi:10.1128/JVI.00417-10. PubMed: 20427538. 20427538 PMC2903297

[44] Fornari F, Gramantieri L, Giovannini C, Veronese A, Ferracin M et al. (2009) MiR-122/cyclin G1 interaction modulates p53 activity and affects doxorubicin sensitivity of human hepatocarcinoma cells. Cancer Res 69: 5761-5767. doi:10.1158/0008-5472.CAN-08-4797. PubMed: 19584283. 19584283

[45] Tsai WC, Hsu SD, Hsu CS, Lai TC, Chen SJ et al. (2012) MicroRNA-122 plays a critical role in liver homeostasis and hepatocarcinogenesis. J Clin Invest 122: 2884-2897. doi:10.1172/JCI63455. PubMed: 22820290. 22820290 PMC3408747

[46] Hsu SH, Wang B, Kota J, Yu J, Costinean S et al. (2012) Essential metabolic, anti-inflammatory, and anti-tumorigenic functions of miR-122 in liver. J Clin Invest 122: 2871-2883. doi:10.1172/JCI63539. PubMed: 22820288. 22820288 PMC3408748

[47] Burns DM, D’Ambrogio A, Nottrott S, Richter JD (2011) CPEB and two poly(A) polymerases control miR-122 stability and p53 mRNA translation. Nature 473: 105-108. doi:10.1038/nature09908. PubMed: 21478871. 21478871 PMC3088779

[48] Lardizábal MN, Nocito AL, Daniele SM, Ornella LA, Palatnik JF et al. (2012) Reference genes for real-time PCR quantification of microRNAs and messenger RNAs in rat models of hepatotoxicity. PLOS ONE 7: e36323. doi:10.1371/journal.pone.0036323. PubMed: 22563491. 22563491 PMC3341372

[49] Norman KL, Sarnow P (2010) Modulation of hepatitis C virus RNA abundance and the isoprenoid biosynthesis pathway by microRNA miR-122 involves distinct mechanisms. J Virol 84: 666-670. doi:10.1128/JVI.01156-09. PubMed: 19846523. 19846523 PMC2798415

[50] Li Y, Masaki T, Lemon SM (2013) miR-122 and the Hepatitis C RNA genome: More than just stability. RNA Biol 10: 919–24. PubMed: 23770926. 23770926 10.4161/rna.25137 PMC3904590

[51] Janssen HL, Reesink HW, Lawitz EJ, Zeuzem S, Rodriguez-Torres M et al. (2013) Treatment of HCV Infection by Targeting MicroRNA. N Engl J Med, 368: 1685–94. PubMed: 23534542. 23534542 10.1056/NEJMoa1209026

[52] McGivern DR, Lemon SM (2011) Virus-specific mechanisms of carcinogenesis in hepatitis C virus associated liver cancer. Oncogene 30: 1969-1983. doi:10.1038/onc.2010.594. PubMed: 21258404. 21258404 PMC3642622

[53] Chen Y, Shen A, Rider PJ, Yu Y, Wu K et al. (2011) A liver-specific microRNA binds to a highly conserved RNA sequence of hepatitis B virus and negatively regulates viral gene expression and replication. FASEB J 25: 4511-4521. doi:10.1096/fj.11-187781. PubMed: 21903935. 21903935 PMC3236624

[54] Wang S, Qiu L, Yan X, Jin W, Wang Y et al. (2012) Loss of microRNA 122 expression in patients with hepatitis B enhances hepatitis B virus replication through cyclin G(1) -modulated P53 activity. Hepatology 55: 730-741. doi:10.1002/hep.24809. PubMed: 22105316. 22105316

[55] Liu WH, Yeh SH, Chen PJ (2011) Role of microRNAs in hepatitis B virus replication and pathogenesis. Biochim Biophys Acta 1809: 678-685. doi:10.1016/j.bbagrm.2011.04.008. PubMed: 21565290. 21565290

[56] Qiu L, Fan H, Jin W, Zhao B, Wang Y et al. (2010) miR-122-induced down-regulation of HO-1 negatively affects miR-122-mediated suppression of HBV. Biochem Biophys Res Commun 398: 771-777. doi:10.1016/j.bbrc.2010.07.021. PubMed: 20633528. 20633528

[57] Liang Y, Shilagard T, Xiao SY, Snyder N, Lau D et al. (2009) Visualizing hepatitis C virus infections in human liver by two-photon microscopy. Gastroenterology 137: 1448-1458. doi:10.1053/j.gastro.2009.07.050. PubMed: 19632233. 19632233

[58] Jung CJ, Iyengar S, Blahnik KR, Ajuha TP, Jiang JX et al. (2011) Epigenetic modulation of miR-122 facilitates human embryonic stem cell self-renewal and hepatocellular carcinoma proliferation. PLOS ONE 6: e27740. doi:10.1371/journal.pone.0027740. PubMed: 22140464. 22140464 PMC3225380

[59] Arai E, Ushijima S, Gotoh M, Ojima H, Kosuge T et al. (2009) Genome-wide DNA methylation profiles in liver tissue at the precancerous stage and in hepatocellular carcinoma. Int J Cancer 125: 2854-2862. doi:10.1002/ijc.24708. PubMed: 19569176. 19569176

[60] Jung JK, Park SH, Jang KL (2010) Hepatitis B virus X protein overcomes the growth-inhibitory potential of retinoic acid by downregulating retinoic acid receptor-beta2 expression via DNA methylation. J Gen Virol 91: 493-500. doi:10.1099/vir.0.015149-0. PubMed: 19828754. 19828754

[61] Hsia CC, Thorgeirsson SS, Tabor E (1994) Expression of hepatitis B surface and core antigens and transforming growth factor-alpha in "oval cells" of the liver in patients with hepatocellular carcinoma. J Med Virol 43: 216-221. doi:10.1002/jmv.1890430304. PubMed: 7523580. 7523580

[62] Pedersen IM, Cheng G, Wieland S, Volinia S, Croce CM et al. (2007) Interferon modulation of cellular microRNAs as an antiviral mechanism. Nature 449: 919-922. doi:10.1038/nature06205. PubMed: 17943132. 17943132 PMC2748825

[63] Wieland S, Thimme R, Purcell RH, Chisari FV (2004) Genomic analysis of the host response to hepatitis B virus infection. Proc Natl Acad Sci U S A 101: 6669-6674. doi:10.1073/pnas.0401771101. PubMed: 15100412. 15100412 PMC404103

[64] Bigger CB, Guerra B, Brasky KM, Hubbard G, Beard MR et al. (2004) Intrahepatic gene expression during chronic hepatitis C virus infection in chimpanzees. J Virol 78: 13779-13792. doi:10.1128/JVI.78.24.13779-13792.2004. PubMed: 15564486. 15564486 PMC533929

[65] Lanford RE, Guerra B, Bigger CB, Lee H, Chavez D et al. (2007) Lack of response to exogenous interferon-alpha in the liver of chimpanzees chronically infected with hepatitis C virus. Hepatology 46: 999-1008. doi:10.1002/hep.21776. PubMed: 17668868. 17668868 PMC2386986

