# Supplementary figures and images for: microRNA-122 Abundance in Hepatocellular Carcinoma and Non-Tumor Liver Tissue from Japanese Patients with Persistent HCV versus HBV Infection
Source: PLoS One. 2013 Oct 9;8(10):e76867. doi: 10.1371/journal.pone.0076867 (PMC3793926; doi:10.1371/journal.pone.0076867)

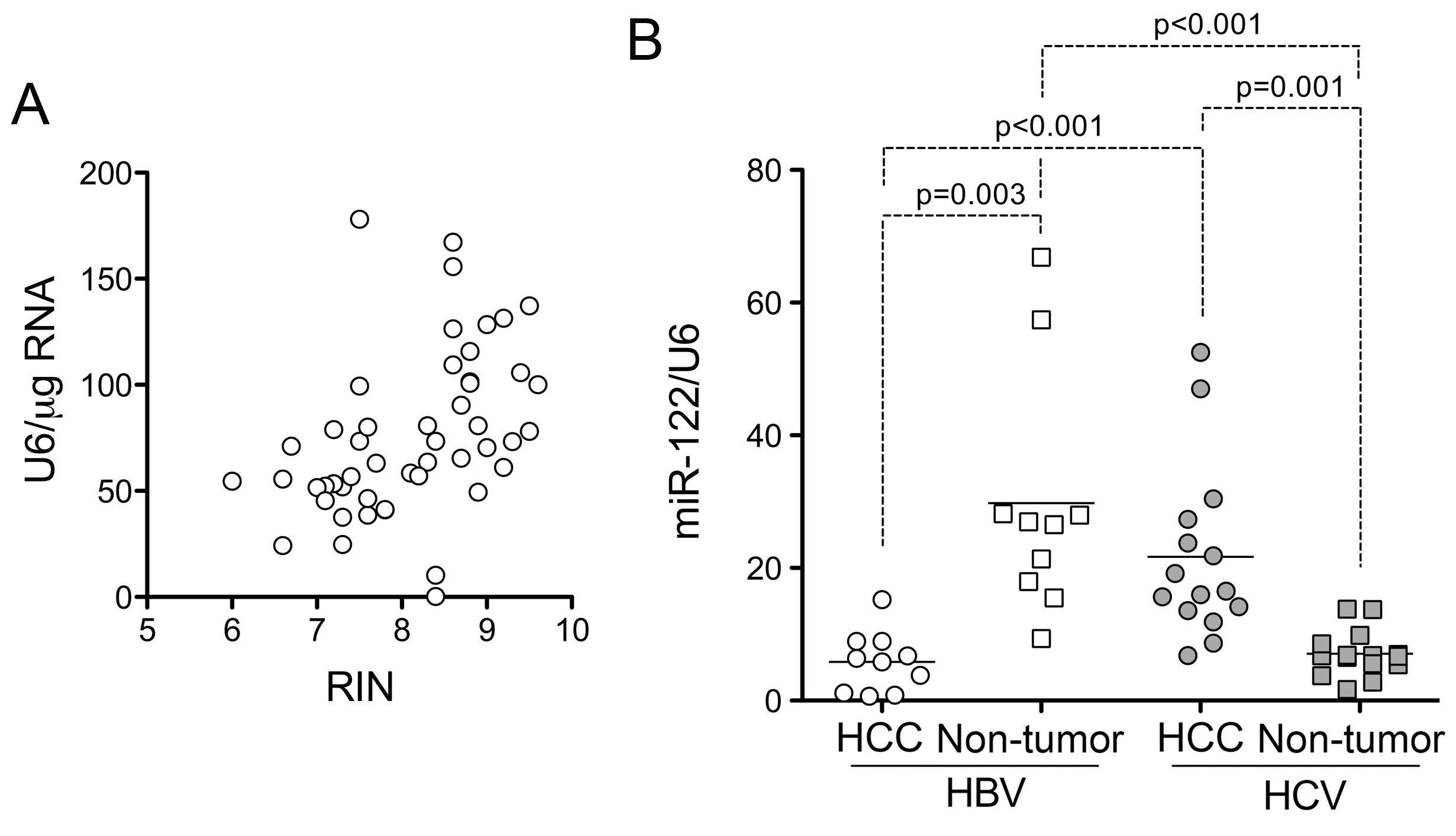

Supplement: Figure S1 — U6 snRNA copy number as a standard for normalization of miR-122 abundance. (A) U6 copy number (relative copy number per µg RNA) plotted as a function of the RNA integrity number (RIN score, on a scale of 1 to 10) determined as described in Methods in the main text. A strong negative correlation exists between U6 copy number and the RIN score: Spearman rs = 0.5216, two-tailed p = 0.0001). (B) miR-122 abundance in HCC and non-tumor tissues from HBV- and HCV-infected subjects, normalized to U6 snRNA copy number. Statistical significance was assessed using paired and unpaired t tests, as described in the main text. (TIF) [file pone.0076867.s001.tif]
